# Effects of Tai Chi on the quality of life, mental wellbeing, and physical function of adults with chronic diseases: Protocol for a single-blind, two-armed, randomised controlled trial

**DOI:** 10.1371/journal.pone.0270212

**Published:** 2022-06-24

**Authors:** Carol Chunfeng Wang, Johnny Lo, Sadie Geraghty, Angela Wei Hong Yang

**Affiliations:** 1 School of Nursing, Midwifery, Health Sciences & Physiotherapy, The University of Notre Dame Australia, Perth, Western Australia; 2 School of Nursing and Midwifery, Edith Cowan University, Perth, Western Australia; 3 School of Science, Edith Cowan University, Perth, Western Australia; 4 School of Health and Biomedical Sciences, RMIT University, Bundoora, Australia; Prince Sattam Bin Abdulaziz University, College of Applied Medical Sciences, SAUDI ARABIA

## Abstract

**Introduction:**

Quality of life (QoL), mental wellbeing, and physical function are often diminished among people with chronic disease. Tai Chi is a moderate form of exercise that may be effective in improving chronic disease management. This protocol paper outlines a trial to determine the therapeutic effects of a Tai Chi program on chronic disease management.

**Methods and analysis:**

This study will be a pilot, interventional, single-blind, two-armed, randomised, parallel, and controlled trial involving a 12-week Tai Chi program for Australian adults. Forty people aged 18 years and older, diagnosed with one or more chronic disease from general community will be recruited. All participants will be randomised to either a 12-week Tai Chi program or a waiting list control group. The Tai Chi program will involve 12 weeks of group Tai Chi sessions, with 45 minutes per session, twice a week. The primary outcome will be QoL as measured by mean scores on the 12-item Short Form Health Survey (SF-12v2) and the EuroQoL (EQ-5D). The secondary outcomes will include anxiety as measured by mean score on the generalised anxiety disorder 7 (GAD-7) survey; depression as measured by mean score on the patient health questionnaire (PHQ-9); work productivity and activity assessment (WPAI:SHP); pain (if any) as measured by mean scores on the visual analogue scale (VAS) and the McGill pain questionnaire (MPQ). These primary and secondary outcomes will be self-administered via two online assessments prior to (T0) and post-intervention (T1). Objective measures as additional secondary outcomes, will also be carried out by the research team including flexibility as measured by the finger to floor distance (FFD); obesity as measured by mean scores on body mass index (BMI); vital signs (blood pressure, heart rate, respiratory rate, temperate, and oxygen saturation) as measured by a blood pressure monitor, tympanic, and pulse oximetry device, and these outcomes will be measured at T0 and T1 in the ECU Holistic Health Research Clinic. People diagnosed with pre-diabetes or diabetes, their glycosylated haemoglobin (HbA1C) and fasting (before breakfast) blood glucose level (BGL) will also be measured via test kits at T0 and T1 in the clinic.

Linear mixed modelling will be conducted to assess changes in outcomes. Statistical significance will be set at an alpha level of 0.05 with a medium effect size. All analyses will be conducted using R version 4.1. Qualitative data will be analysed using template thematic analysis.

**Ethics and dissemination:**

Ethical approval has been obtained from the Edith Cowan University (ECU) Human Research Ethics Committee (2021-03042-WANG). Research findings will be disseminated to the public, health professionals, researchers, and healthcare providers through conference presentations, lay summaries, and peer-reviewed publications. This study will provide an updated evidence on a safe, sustainable, and inexpensive non-pharmacological approach in the management of chronic disease, the number one burden of disease in Australia.

**Trial registration:**

**Trial registration number:** ACTRN12622000042741p.

## Introduction

According to Australian National Health Survey in 2018, almost half of the Australians (47.3%) had one or more chronic diseases. Of these, mental and behavioural conditions were the most common, reported by 1 in 5 adults (20.1%), followed by back issues (16.4%), arthritis (15.0%), asthma (11.2%), diabetes mellitus (4.9%), heart, stroke and vascular disease (4.8%), osteoporosis (3.8%), chronic obstructive pulmonary disease (COPD) (2.5%), cancer (1.8%), and kidney disease (1.0%) [1]. In 2018, 1 in 5 Australian adults (20%) were living with two or more chronic diseases; 1 in 2 (51%) experienced a hospitalisations related to a chronic disease; and nearly 9 in 10 (89%) of deaths were associated with chronic disease [2].

Chronic diseases are becoming increasingly common and their social and economic consequences impact on peoples’ quality of life (QoL). Although its definition is still evolving, QoL, by its very nature, is “idiosyncratic to the individual, but intuitively meaningful and understandable to most people” [3]. QoL consists of a physical aspect (e.g., health, diet, protection against pain and disease) and psychological aspect (e.g., stress, worry, pleasure and other positive or negative emotions). It is therefore impossible to measure directly. However, we can assume that the higher average level of diet, shelter, safety, as well as freedoms and rights a general population has, the better overall QoL said population experiences [4].

Chronic pain, for example, in 2020, affected more than 3.4 million Australians, and 43% of them had lived with the condition for more than five years. Cost caused by the reduction in QoL in chronic pain was estimated in 2020 at $46.66 billion. The total financial costs of chronic pain were $144.1 billion in 2020, and is expected to rise to $215.6 billion by 2050 [5]. Where pain is managed, medications are used in close to 70% of general practitioner (GP) consultations [5]. Best practice does not support long term medication use for chronic pain management due to the side effects. Chronic diseases are the priority for action in Australia’s health sector. A non-pharmaceutical therapy such as Tai Chi could serve as an adjunct to standard medical care to elevate the burden of these diseases.

Tai Chi, an ancient Chinese practice, has become widely recognised as one of the most popular ways to improve both physical and mental health. Several studies demonstrated that Tai Chi has many health benefits, such as decreased heart rate, decreased blood pressure, lowered lipid levels, decreased levels of stress hormones, enhanced immune function, and improved physical and psychological wellbeing [6,7]. However, more consolidated evidence for the role of Tai Chi in chronic disease management is required. This study, therefore, aims to evaluate the therapeutic effects of a 12-week Tai Chi program as a non-pharmacological approach in chronic disease management. We hypothesise that QoL, physical function, and mental wellbeing in Tai Chi group will be better than the waiting list control group. We hope that the results of our research can be used to inform how to better manage chronic diseases clinically. There are no foreseeable risks associated with this research project.

## Methods and analysis

### Study design

This study will be a pilot, interventional, single-blind (assessors blind to group allocation), two-arm, randomised, parallel, and controlled trial on the therapeutic effect of a 12-week Tai Chi program for Australia adults. The trial protocol will be reported adhering to the Standard Protocol Items: Recommendations for Interventional Trials (SPIRIT) reporting template [8].

### Sample size estimation

Calculation of sample size is based on the desired effect size, level of significance, power and expected attrition rate. A study [9] reported a significant difference (t (144) = 5.761, p<0.001) in the total QoL between the control (-0.13 ± 10.92 pre-to-post difference) and intervention group (+8.86 ±11.13 pre-to-post difference), which corresponded to a large effect (Cohen’s d = 0.91). Within the QoL metric, the emotion well-being component saw the smallest, but still a medium effect (Cohen’s d = 0.58). Given this, we estimated the sample size using G*Power Version 3.1.10 (University of Düsseldorf, Germany) [10], based on a mixed model design (2 groups and 2 time-points) to detect at least a medium Group × Time interaction effect (Cohen’s *f* = 0.25) for QoL (therapeutic effect) at an α of 0.05 (two-tailed) and an 80% power and 5% level of significance. The required sample size is 34 participants. Assuming a drop-out rate of 15%, the adjusted required sample size is 40, or 20 participants for each study group. The sample size will suffice for all QoL components, and as well as the total QoL.

### Participants

Participants will be recruited from the community by the research team at ECU via mass mailings, social media advertising, flyers, and word of mouth referrals. After expressing an interest in participating in this trial, each potential participant will be contacted and screened for inclusion by the investigators and receive further details of the study. Inclusion criteria consist of (1) adults aged 18 years and older, (2) diagnosed with one or more chronic disease (e.g., pain, diabetes, cancer), (3) having no physical and mental limitations prohibiting exercise, and (4) willing and able to consent for participation in the trial. People who are pregnant or with a serious health condition that incapable of completing 12-week Tai Chi exercise will be ineligible to participate.

### Intervention

Participants in the intervention group will receive a Tai Chi program that will be led by a qualified academic at ECU, the program will involve 12 weeks of group Tai Chi sessions, with 45 minutes per session, twice a week.

### Comparison

Participants in the waiting list control group will receive usual care (e.g., other exercise or medication).

### Randomisation and blinding

Sequence number of each participant will be computer generated using a randomised block approach. The block sizes will not be disclosed to ensure concealment. The allocation will be performed by an independent, blinded statistician. The randomisation list will only be kept by the researcher who performed the intervention. Participants will be randomly assigned to one of the two arms: (1) Tai Chi group or (2) waiting list control group. Outcome assessors and team members who perform data entry and data analysis will be blinded to the randomisation.

### Study procedures

**Fig 1:**
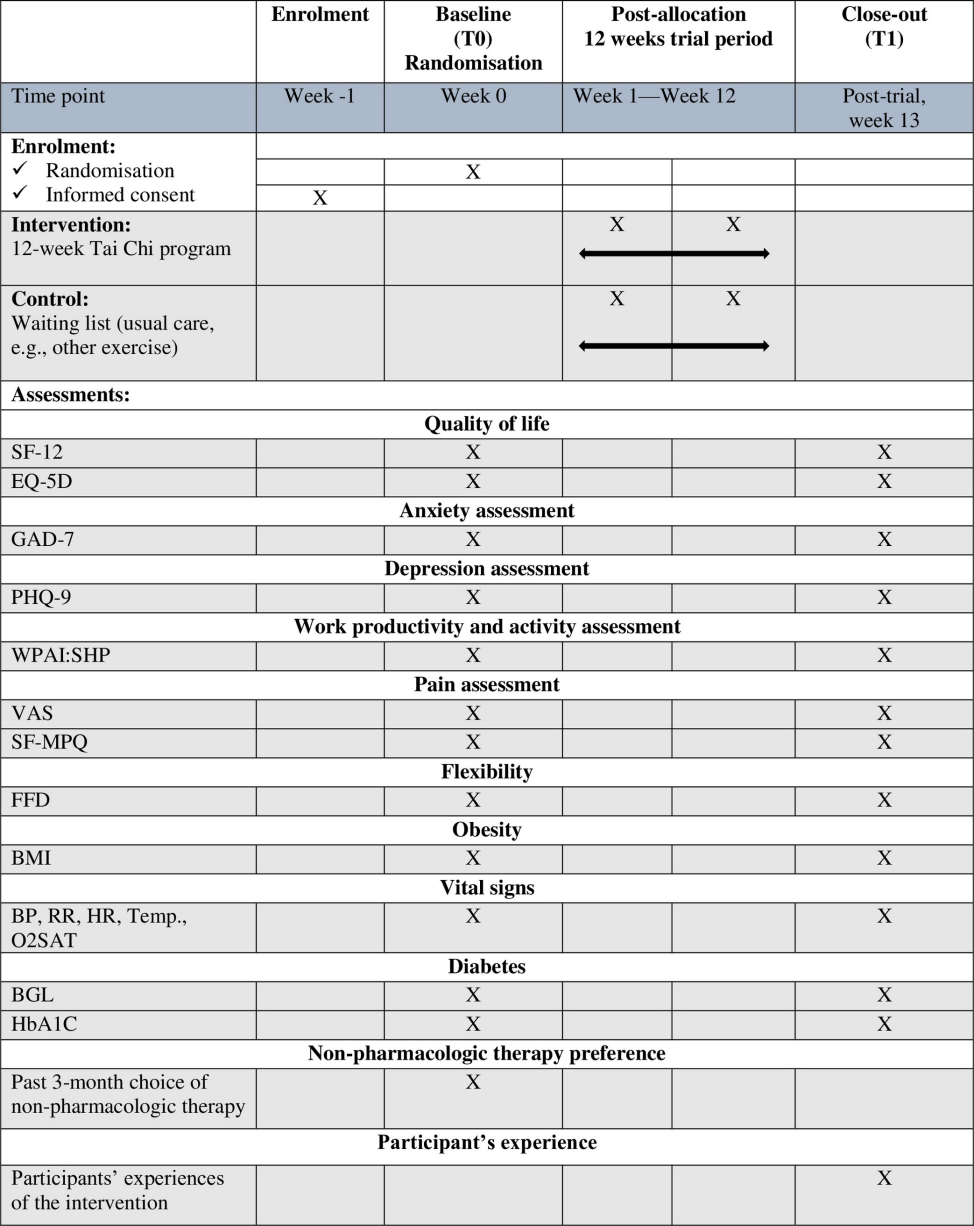
The schedule of enrolment, interventions, and assessments. Note: BP, RR, HT, Temp., O2SAT = blood pressure, respiratory rate, heart rate, temperate, oxygen saturation; BMI = Body mass index; BGL = blood glucose level; EQ-5D = EuroQoL (EQ-5D); FFD = Finger to Floor Distance; HbA1C = Glycosylated haemoglobin; GAD-7 = Generalised Anxiety Disorder 7; PHQ-9 = Patient Health Questionnaire; SF-12 = 12-item Short Form Health Survey; SF-MPQ = Short-Form McGill Pain Questionnaire; VAS = visual analogue scale; WPAI:SHP = Work Productivity and Activity Impairment Questionnaire for Specific Health Problem V2.0.

All participants will complete a battery of self-report questionnaires at baseline (T0) and at the end of intervention (week 12) (T1). These questionnaires should not take more than 15 minutes for both T0 and T1. A set of physical measurements will also be taken at T0 and T1. The physical measurements should not take more than 20 minutes for both T0 and T1. Fig 1 summarises the schedule of enrolment, interventions, and assessments.

### Public involvement

A consumer representative will be involved in this study. Consumer involvement in this project aims to help consider implications of the research and how it will lead to practical benefits for the chronic disease community in the future [11]. The consumers will advise on the study design, how to best connect with potential study participants, conduct interpretation of the findings and dissemination of findings.

### Concomitant care

The 12-week Tai Chi program is an ongoing initiative. Participants will remain eligible to access the program. If they wish, they can continue attend the Tai Chi classes for free after their completion of the study.

### Ethics and endpoint

#### Ethics and dissemination

The study will be conducted following the National Statement and the Australian Code for the Responsible Conduct of Research, 2018 (the ‘Research Code’), and ethical approval has been obtained from ECU’s Human Research Ethics Committee (No. 2021-03042-WANG). The Participant Information Letter will be provided to all participants to explain the study, including the purpose and procedures, the voluntary nature of participation, and the option to withdraw at any time. Participants will also be guaranteed confidentiality and secured data storage. Any adverse events arising will be reported and managed by the investigators. Data will be securely stored in ECU’s security location, and no unauthorised persons will have access to the collected data. The investigator will supply the Ethics Committee on request with any required background data from the study documentation or clinic records. In case of special problems and/or governmental queries or requests for audit inspections, it is also necessary to have access to the complete study records, where participant confidentiality is protected.

The research findings will be shared in various forms to engage broader audiences, including at national and international conferences presentations, in open-access peer-reviewed journal publications, and at local community workshops with healthcare professionals.

#### Outcome measures

The **primary outcome** will be QoL as measured by mean scores on the 12-item Short Form Health Survey (SF-12) [12,13] and the EuroQoL (EQ-5D) [14]. This outcome will be obtained via two online surveys: at baseline (T0) and post intervention (T1).

The **secondary outcomes** will include anxiety as measured by mean score on generalised anxiety disorder 7 survey (GAD-7) [15]; depression as measured by mean score on the patient health questionnaire (PHQ-9) [16]; work productivity and activity assessment survey (WPAI:SHP) [17,18]; pain as measured by mean scores on the visual analogue scale (VAS) [19,20] and the McGill pain questionnaire (MPQ) [21–24]. These outcomes will be measured via two online assessments (T0 and T1). Questions on participants’ non-pharmacologic therapy preferences and experiences of participating in the trial will also be included, and measured at T0 and T1, respectively.

In addition to these self-reported questionnaires, objective measures will also be carried out by the research team at ECU Holistic Health Research Clinic. These measures include flexibility as measured by the finger to floor distance (FFD); obesity as measured by mean scores on body mass index (BMI); vital signs (blood pressure, heart rate, respiratory rate, temperate, and oxygen saturation) as measured by a blood pressure monitor, tympanic, and pulse oximetry device. These outcomes will be measured prior to (T0) and post-intervention (T1). People diagnosed with pre-diabetes or diabetes, their HbA1C and fasting BGL will also be measured at the clinic via test kits at T0 and T1.

*Quality of life assessment*. **SF-12.** The 12-item Short Form Health Survey (SF-12) is a self-reported outcome measure assessing the impact of health on an individual’s everyday life and their QoL [12,13], across eight domains, including (1) Limitations in physical activities because of health problems; (2) Limitations in social activities because of physical or emotional problems; (3) Limitations in usual role activities because of physical health problems; (4) Bodily pain; (5) General mental health (psychological distress and well-being); (6) Limitations in usual role activities because of emotional problems; (7) Vitality (energy and fatigue); and (8) General health perceptions. The SF-12 is a validated tool [25–27]. Scores on these eight domains are aggregated to form two final components: physical and mental wellbeing scores. An algorithm is used to generate the two components for comparison to normative data: the mean score is set to 50, scores >50 indicate better physical or mental health than the mean, whereas scores <50 indicate worse physical or mental health than the mean [12,13].

**EQ-5D.** The EuroQoL (EQ-5D) is one of the most used generic health-related QoL questionnaire in adults [14]. It consists of 5 questions that assess functions in 5 dimensions (mobility, self-care, usual activities, pain/discomfort, and anxiety/depression) with 3 possible levels of disability (3, severe; 2, moderate; 1, none). The sum of the responses to each question (11111–33333) with the states of death and unconscious offers a descriptive system of 245 different health states. Each health state could be transformed and ranked in a single score (utility) expressing the QoL Adjusted-Years (QALY) which is commonly used to make evidence-based decisions in analyses of cost-effectiveness. Therefore, the EQ-5D can be used for health outcomes studies and economic analyses. EQ-5D can be used in clinical trials, in population health surveys, in routine outcome measurement and many other types of studies where a generic measure of health status is useful. Extensive research has shown EQ-5D to be valid, reliable, and responsive [28,29].

*Anxiety assessment*. **GAD-7.** The Generalised Anxiety Disorder 7 (GAD-7) is a gold-standard measurement tool for generalised anxiety disorder [15]. It is a quick, user-friendly, concise, and self-administered screening and diagnostic tool. GAD-7 is calculated by assigning scores of 0, 1, 2, and 3 to the response categories of “not at all”, “several days”, “more than half the days”, and “nearly every day”, respectively. GAD-7 total score, the sum of the response scores for the 7 items, ranges from 0 to 21. Scores of 5, 10, and 15 represent cut-off points for mild, moderate, and severe anxiety, respectively.

*Depression assessment*. **PHQ-9.** The Patient Health Questionnaire (PHQ-9) is a self-administered diagnostic instrument for depression severity [16]. It is calculated by assigning scores of 0, 1, 2, and 3 to the response categories of “not at all”, “several days”, “more than half the days”, and “nearly every day”, respectively. PHQ-9 total score for the nine items ranges from 0 to 27. Scores of 5, 10, 15, and 20 represent cut-off points for mild, moderate, moderately severe, and severe depression.

*Work productivity and activity assessment*. **WPAI: SHP.** The Work Productivity and Activity Impairment Questionnaire for Specific Health Problem V2.0 (WPAI: SHP) [17,18] is a 6-item questionnaire that evaluates self-reported productivity and activity during the past week. It includes subscales for absence from work (absenteeism), lost productivity while at work (presenteeism), overall work impairment, and the effects on non-work-related activities. A higher subscale value (0–100%) indicates greater work or activity impairment [17,18].

*Pain assessments*. **VAS and SF-MPQ.** The visual analogue scale (VAS) and the Short-Form McGill Pain Questionnaire (SF-MPQ) are commonly used clinically to assess SNP [30]. VAS is a reliable, validated tool with adequate sensitivity [19,20] that is often used to assess pain intensity. Pain is a subjective experience; therefore, it cannot simply be objectively measured but must also be assessed. Multi-dimensional assessment tools can evaluate multiple aspects of pain, such as sensation, mood, and intensity. The McGill Pain Questionnaire (MPQ) is the most well-known and popular multi-dimensional pain assessment tool. However, with 78 pain descriptors, it is often clinically impractical. The Short-Form McGill Pain Questionnaire (SF-MPQ) was published in 1987, consisting of 15 pain descriptors: 11 that assess the sensory dimension of pain and 4 that assess the affective dimension of pain. Descriptors are rated on an intensity scale of none (= 0), mild (= 1), moderate (= 2), and severe (= 3). Three pain scores are derived from the sum of the intensity rank values of the chosen sensory, affective, and total descriptors [30]. This questionnaire is used to measure the quality (i.e., using words to describe the pain, such as ‘sharp’, ‘dull’, ‘stabbing’, ‘burning’, ‘crushing’, ‘throbbing’, ‘nauseating’, ‘shooting’, ‘twisting’ or ‘stretching’) as well as the intensity of pain [21–24].

*Flexibility*. Finger to Floor Distance (FFD) will be measured with the patient bending forward maximally with knees straight. We will use a ruler to assess flexibility by measuring the FFD. Two measurements will be made and averaged.

*Obesity*. Body mass index (BMI) is an internationally recognised standard for classifying overweight and obesity in adults. BMI is calculated by dividing a person’s weight in kilograms by the square of their height in metres. A BMI of 25.0–29.9 is classified as overweight but not obese, while a BMI of 30.0 or over is classified as obese. A BMI of greater than 35.0 is classified as severely obese [31].

*Vital signs*. Vital signs indicate the status of the body’s vital functions. These measurements are taken to assess the general physical health: body temperature (Temp), heart rate (HR), respiratory rate (RR), blood pressure (BP), and oxygen saturation (SpO2) [32].

#### Body temperature (Temp)

Temperature is how hot or cold a person is, and a normal range is considered to be between 36.6 to 37.5 degrees Celsius. Temperature is measured via a tympanic device.

#### Heart rate (HR)

HR is the number of heart beats per minute. The normal range is between 60 to 100 beats per minute. This result can be influenced by fitness level, age, illness, and emotion.

#### Respiratory rate (RR)

RR is the number of breaths a person takes per minute. It is measured through watching the rise and fall of the chest and counting breaths for a full minute. The normal limit for an adult RR is between 12 and 20 breaths per minute. A person’s RR is influenced by factors such as resting or moving.

#### Blood pressure (BP)

BP consists of systolic pressure (the first number) indicates the pressure of blood within the arteries during a contraction of the left ventricle of the heart. The diastolic reading (the second number) indicates the pressure within the arteries when the heart is at rest. According to the Heart Foundation of Australia, as a general guide, BP can be classified as ‘optimal’ (<120/80), ‘normal’ (120/80-129/84), and ‘high-normal’ (130/85-139/89). High BP is further classified as mild (140-159/ 90–99), moderate (160-179/ 100–109) or severe (≥180/110).

#### Oxygen saturation (SpO2)

SpO2 estimates the level of oxygen blood carries. A pulse oximetry device is used to present the measurement with a percentage. If the red blood cells contain 95% oxygenated haemoglobin, the SpO2 would be 95%. Normal pulse oximeter readings usually range from 95 to 100 percent. Values under 90 percent are considered low and indicate the need for supplemental oxygen. This condition is often referred to as hypoxemia, and its symptoms include severe shortness of breath, increased heart rate and chest pain.

*Blood glucose level (BGL)*. One of the aims of diabetes management is to keep BGL within a target range via balanced food intake, appropriate physical activity, healthy lifestyle choice and diabetes medications. Keep BGL within a target range can reduce a risk of developing diabetes related complications. The ranges will vary depending on the individual and their circumstances. According to Diabetes Australia [33], the target range for fasting BGL is between 4 to 6 mmol/L.

*Glycosylated haemoglobin (HbA1C) check*. The HbA1C shows an average of one’s BGL over the past 3 months and is checked every 3–6 months. Different from BGL, HbA1C provides an overall picture of one’s BGL management. The goal for most people with diabetes will be in the 6.5–7 percent (48-53mmol/mol) range [33].

### Statistical analysis

Descriptive statistics for continuous variables will be described by mean and standard deviation (SD) for normal data, and by median and interquartile range (IQR) for non-normal data. Categorical data will be summarised by frequencies and proportions. The outcomes will be assessed following intention-to-treat principles. Linear mixed modelling with unstructured covariance matrix will be conducted to assess changes in outcomes throughout the study. This modelling approach allows for the inclusion of missing data in an intention-to-treat analysis without imputations (e.g., last-observation-carried-forward). Post-hoc tests will be conducted on all pairwise comparisons. The analysis will be adjusted for potential confounding factors such as age, gender, education levels and any other potentially relevant variables where data are available. The corrected Akaike Information Criterion (AICc) will be used to assess model fit when covariates are added to the model. Normality assumptions will be assessed using the Shapiro-Wilk test. If required, non-linear transformations such as the square root and log-transformations, will be applied to normalise the data. Statistical significance will be set at an alpha level of 0.05. Effect sizes, defined by partial eta squared, will be reported and interpreted, with 0.01, 0.06 and 0.014, respectively, identified as small, medium and large effects [34]. All analyses will be conducted using R version 4.1.

The qualitative data collected via open-ended questions from the post-intervention online assessment will be used to help explain or elaborate on the quantitative data. Qualitative data will be analysed using template thematic analysis. Template thematic analysis uses ‘a priori’ code frames to analyse and report on the data [35]. The initial skeleton code frame is often formulated from the questions asked of participants and then built upon during analysis in an iterative process.

## Implication of the study

Evidence has shown that Tai Chi can improve both physical and mental aspects for people with chronic disease [36]. It is safe, easy to learn, in expensive, and low intensity exercise with increasing popularity worldwide [37]. Findings from the present study will advance knowledge on treatment options with a potential to best manage chronic disease. Our recommendations will inform healthcare’s policy and priorities in the area. The findings will provide insight into the potential of alternative management strategies to support improving QoL, financial concerns, decision making, and ultimately help people with chronic disease to live well.

If the intervention improves some of the chronic disease symptoms, the current study will build research capacity and increase public awareness, improve the quality and appropriateness of patient care, and enhance the health professional-patient relationship. The developed evidence-based self-managing chronic disease guideline will empower patients’ self-care and improve their health status.

### Conditions for protocol modifications

Protocol modifications will be undertaken by the principal investigator and will be submitted to the appropriate Independent Ethics Committee or Institutional Review Board for information and approval in accordance with local requirements, and to Regulatory Agencies if required. Approval will be awaited before any changes are implemented, or when the change(s) involves only logistical or administrative aspects of the trial (e.g., change in monitors, change of telephone numbers).

## Supporting information

S1 File SPIRIT checklist with page reference numbers. (DOC)

S2 File Trial protocol for ethics application. (DOCX)
