# Supplementary material for: Effects of Tai Chi on the quality of life, mental wellbeing, and physical function of adults with chronic diseases: Protocol for a single-blind, two-armed, randomised controlled trial
Source: PLoS One. 2022 Jun 24;17(6):e0270212. doi: 10.1371/journal.pone.0270212 (PMC9231742; doi:10.1371/journal.pone.0270212)
Supplement: S2 File — (DOCX) [file pone.0270212.s002.docx]

| 1. Project synopsis |  |
| --- | --- |
| **Name of the Funding Source:** TBC | **Protocol Number:** V.5 |
| **Study Title:** Effects of Tai Chi on the quality of life, mental wellbeing, and physical function of adults with chronic diseases: protocol for a single-blind, two-armed, randomised controlled trial | |
| **Short Title:** Tai Chi for chronic disease management: a trial protocol | |
| **Planned Study Sites:** ECU Sport Centre and or local communities, Perth, Australia | |
| **Number of Participants:** Forty adults aged 18 years and older, diagnosed with one or more chronic disease (e.g., pain, diabetes, cancer), will be included in this study. | |
| **Chief Investigator:**  Dr Carol Wang  School of Nursing and Midwifery  Edith Cowan University  Western Australia, 6027 | |
| **Study Period:** 03/01/2022—02/03/2032  Date of first participant enrolment: 01/06/2022 Estimated date of last participant completed: 02/12/2023 | |
| **Objectives:**  The overarching aim of this study is to provide compelling evidence on the therapeutic efficacy of the Tai Chi/Qigong—Body & Mind Medicine program chronic disease management | |
| **Study Design:** This is a single-blind (assessors blind to group allocation), double-armed, randomised controlled trial on the therapeutic effect of a 12-week Tai Chi/Qigong exercise for Australia adults | |
| **Study Criteria**  **Key inclusion criteria**  Adults who are aged between 18 years and older willing and able to consent  **Key exclusion criteria**  Inclusion criteria consist of (1) adults aged 18 years and older, (2) diagnosed with one or more chronic disease (e.g., pain, diabetes, cancer), (3) having no physical and mental limitations prohibiting exercise, and (4) willing and able to consent for participation in the trial. People who are pregnant or with a serious health conditions that incapable of completing 12-week Tai Chi exercise will be ineligible to participate. | |
| **Intervention and exposure:**  Intervention group:  Participants will receive a 12-week Tai Chi/Qigong exercise from a licensed acupuncturist at ECU Holistic Health Research Clinic within ECU Sport Centre. 45 minutes per session, three times a week for twelve weeks. Although the program runs three times per week, but participants are expected to attend any of two sessions within that week. At the beginning (baseline, T0) and the end of receiving twelve weeks Tai Chi/Qigong exercise (post trial, T1), participants will be asked to complete the baseline (T0) and the post-trial (T1) online questionnaires. The researchers will also conduct a set of objective assessments in the clinic at T0 and T1.  Control group:  Waiting list control with usual care (e.g., other exercise) over the 12-week trial period.  Participants in this group will be informed via email that they are in the waiting list group but will be asked to complete the baseline (T0) and the post-trial (T1) online questionnaires. The researchers will also conduct a set of objective assessments in the clinic at T0 and T1. Participates in this group will join the next batch training in 12 weeks’ time as the Tai Chi/Qigong—Body & Mind Medicine program is an ongoing activity. | |
| **Outcome measures and time points:**  The outcomes are QoL as measured by mean scores on the 12-item Short Form Health Survey (SF-12) and the EuroQoL (EQ-5D); anxiety as measured by mean scores on generalized anxiety disorder 7 (GAD-7); depression as measured by mean scores on the patient health questionnaire (PHQ-9); work productivity and activity assessment (WPAI:SHP) questionnaire; pain as measured by mean scores on the visual analogue scale (VAS), and the McGill pain questionnaire (MPQ). These outcomes will be measured via two online surveys prior to (T0) and post-intervention (T1). Questions on participants’ non-pharmacologic therapy preferences and experiences of participating in the trial will also be included, and measured at T0 and T1, respectively.  In addition to these self-reported questionnaires conducted via online surveys, objective measures will also be carried out by the research team at ECU Holistic Health Research Clinic, which is in the university’s Sport Centre where the Tai Chi/Qigong training program take place. These measures include flexibility as measured by the finger to floor distance (FFD); obesity as measured by mean scores on body mass index (BMI); Vital signs (blood pressure, heart rate, respiratory rate, temperate, and oxygen saturation) as measured by a blood pressure monitor, tympanic, and pulse oximetry device. These outcomes will be measured prior to (T0) and post-intervention (T1). People diagnosed with pre-diabetes or diabetes, their HbA1C and fasting BGL will also be measured via test kits at T0 and T1. | |
| **Sample Size:** Based on previous Tai Chi/Qigong exercise intervention studies, n = 20 participants will be required per group (total n=40) for this study. | |

Element 1

**Aims or Questions**

Project title: Impact of Tai Chi/Qigong—Body & Mind Medicine program on Australian adults’ health

The overarching aim of this study is to provide compelling evidence of the therapeutic efficacy of the Tai Chi/Qigong—Body & Mind Medicine program for chronic disease management.

**Benefit of exploring these research questions**

Several studies demonstrated that Tai Chi/Qigong has many health benefits, such as decreased heart rate, decreased blood pressure, lowered lipid levels, decreased levels of stress hormones, enhanced immune function, and improved physical wellbeing and psychological wellbeing. However, there is no consolidated conclusion for Tai Chi/Qigong and more studies in this field are required to provide updated evidence. Therefore, this study aims to address this knowledge gap.

We hypothesize that Quality of life (QoL) in Tai Chi/Qigong group is better than the control group (usual care). We hope that the results of our research can be used to inform our knowledge about how to better manage chronic diseases that many Australians are suffering.

There are no foreseeable risks associated with this research project.

**Outline the design of this research; its methods and details of the instruments to be used to collect the data, including psychometric properties if applicable.**

**Study design:**

This is a single-blind (assessors blind to group allocation), double-armed, randomised controlled trial on the therapeutic effect of a 12-week Tai Chi/Qigong exercise for Australia adults

**Study type:**

Interventional study

**PICOs:**

**P:** Adults aged 18 years and older will be recruited for this trial

**I:** 12-week Tai Chi/Qigong exercise + usual care

**C:** Waiting list control with usual care (e.g., other exercise)

**O:**

The primary outcome is quality of life as measured by mean scores on the 12-item Short Form Health Survey (SF-12) and the EuroQoL (EQ-5D). This outcome will be obtained via two online surveys: at baseline (T0) and post intervention (T1).

The secondary outcomes will include anxiety as measured by mean scores on generalized anxiety disorder 7 (GAD-7); depression as measured by mean scores on the patient health questionnaire (PHQ-9); work productivity and activity assessment (WPAI:SHP); pain as measured by mean scores on the visual analogue scale (VAS) and the McGill pain questionnaire (MPQ). These outcomes will be measured via two online surveys at T0 and T1. Questions on participants’ non-pharmacologic therapy preferences and experiences of participating in the trial will also be included, and measured at T0 and T1, respectively. Flexibility as measured by the finger to floor distance (FFD); obesity as measured by mean scores on body mass index (BMI); blood pressure (BP), and oxygen saturation (SpO2) as measured by a pulse oximetry device will also be carried out by the researchers, and these outcomes will be measured before the intervention (T0) and post intervention (T1).

**S:** This is a single-blind (assessors blind to group allocation), double-armed, randomised controlled trial on the therapeutic effect of a 12-week Tai Chi/Qigong exercise for adults.

**Key inclusion exclusion criteria**

Inclusion criteria consist of (1) adults aged 18 years and older, (2) diagnosed with one or more chronic disease (e.g., pain, diabetes, cancer), (3) having no physical and mental limitations prohibiting exercise, and (4) willing and able to consent for participation in the trial. People who are pregnant or with a serious health conditions that incapable of completing 12-week Tai Chi exercise will be ineligible to participate.

**Intervention and exposure:**

**Intervention group:**

In addition to usual care, participants will receive a 12-week Tai Chi/Qigong exercise from a licensed acupuncturist at ECU Holistic Health Research Clinic within the sport centre. 45 minutes per session, three times a week for twelve weeks. Although the program runs three times per week, but participants are expected to attend any of two sessions within that week. At the end of twelve weeks receiving the Tai Chi/Qigong exercise, participants will receive an online link to complete the post-intervention questionnaires. The researchers will also complete the physical assessments in the clinic.

**Control group:**

Waiting list non-intervention but usual care (e.g., other exercise) over the 12-week trial period.

Participants in this group will be informed via email that they are in the no intervention waiting list group but will be emailed an online link to complete the post-intervention questionnaire and invited to the clinic to complete other physical assessments.

Participates in the non-intervention waiting list group will join the next batch as the Tai Chi/Qigong—Body & Mind Medicine program is an ongoing activity.

**How the planned methods achieve the aim or research questions**

The primary outcome is quality of life as measured by mean scores on the 12-item Short Form Health Survey (SF-12) and the Quality-of-life scale (QOLS). This outcome will be obtained via two online surveys: at baseline (T0) and post intervention (T1).

The secondary outcomes will include anxiety as measured by mean scores on generalized anxiety disorder 7 (GAD-7); depression as measured by mean scores on the patient health questionnaire (PHQ-9); work productivity and activity assessment (WPAI:SHP); pain as measured by mean scores on the visual analogue scale (VAS) and the McGill pain questionnaire (MPQ). These outcomes will be measured via two online surveys at T0 and T1. Questions on participants’ non-pharmacologic therapy preferences and experiences of participating in the trial will also be included, and measured at T0 and T1, respectively. Flexibility as measured by the finger to floor distance (FFD); obesity as measured by mean scores on body mass index (BMI); blood pressure (BP), and oxygen saturation (SpO2) as measured by a pulse oximetry device will also be carried out by the researchers, and these outcomes will be measured before the intervention (T0) and post intervention (T1).

Statistical analysis:

Descriptive statistics for continuous variables will initially be described by mean and standard deviation (SD) for normal data, and by median and interquartile range (IQR) for non-normal data. Categorical data will be summarised by frequencies and proportions. The primary and secondary outcomes will be assessed following intention-to-treat principles. Linear mixed modelling with unstructured covariance matrix will be conducted to assess changes in outcomes throughout the study. This model allows for the inclusion of missing data in an intention-to-treat analysis without imputations (e.g., last-observation-carried-forward). Post-hoc tests will be conducted on all pairwise comparisons. The analysis will be adjusted for potential confounding factors such as age, gender, education levels and any other potentially relevant variables where data are available. The corrected Akaike Information Criterion (AICc) will be used to assess model fit when covariates are added to the model. Normality assumptions will be assessed using the Shapiro-Wilk test. If required, non-linear transformations such as the square root and log-transformations, will be applied to normalise the data. Statistical significance will be set at an alpha level of 0.05. False discovery rate (FDR). Corrections will be applied to all analysed outcomes to account for multiple comparisons. Effects sizes, defined by partial eta squared, will be reported and interpreted, with 0.01, 0.06 and 0.014, respectively, identified as small, medium and large effects (43). All analyses will be conducted using R version 4.1.

The qualitative data collected via open-ended questions across the two online surveys will be used to help explain or elaborate on the quantitative data. Qualitative data will be analysed using template thematic analysis. Template thematic analysis uses ‘a priori’ code frames to analyse and report on the data. The initial skeleton code frame is often formulated from the questions asked of participants and then built upon during analysis in an iterative process.

Element 2

**Recruitment**

**How will participants be identified and initially contacted including screening processes?**

The snowballing process and public advertisement can identify potential participants. People interested in participating in the study will be encouraged to contact the research team via email for an eligibility check.

**How will formal recruitment of potential participants be conducted?**

The study will involve providing participants with a 12-week Tai Chi/Qigong exercise.

Participants who fulfil our inclusion and exclusion criteria will be assigned with a unique identification number and provided with a Participant Information Letter outlining what will be involved in the trial and an online survey starting with a consent form. People who gave consent will complete the online survey after ticking a box under the online consent form.

Participants who completed the online survey will be invited to ECU sports centre where the 12-week Tai Chi/Qigong exercise take place, the researcher team will carry out the baseline physical assessments (e.g., FFD, BMI, BP, and SpO2).

Following the completion for the baseline measurements, the participants will be assigned to either a control or treatment group, with the assistance of a blinded statistician, via a randomised block design. This ensures the sample number in each group remain relatively similar through the recruitment process until the quota is reached.

Element 3

**Consent**

**Informed consent procedure (including justification for a Waiver of Consent, opt out consent or deception strategies)**

Participant consent form and Participant Information Letter are attached.

Element 4

**Collection, Use and Management of Data and Information**

**Project dates**

**03/01/2022—02/03/2032**

**Data collection method and location including who will be responsible for the data collection**

Participation in this research project is voluntary. If one does not wish to take part, they do not have to. If participant decided to take part and later changed their mind, they are free to withdraw from the project at any time. If they decide to withdraw from the project after the data has been analysed, we will not be able to remove the individual data as this cannot be identified.

If participant do decide to take part, they will be given the Participant Information letter and consent form to sign. They decision to take part, or to take part and later withdraw, will not affect they relationship with the research team and any staff within the School of Nursing and Midwifery at ECU.

By sign the consent form, they consent to the research team collecting and using personal information about the participant for the research project. Any information obtained in connection with this research project that can identify participant will remain confidential. When all data collected are returned to the research team and automatically de-identified. They information will only be used for the purpose of this research project and it will only be disclosed with their permission, except as required by law.

It is anticipated that the results of this research project will be published and/or presented in a variety of professional forums. In any publication and/or presentation, the information will be provided in such a way that participants cannot be identified, except where requested for specific reasons, and then they will be asked to provide written consent.

In accordance with relevant Australian and/or Western Australian privacy and other relevant laws, participants have the right to request access to the information about them that is collected and stored by the research team. They also have the right to request that any information that they disagree to be corrected. They are encouraged to inform the research team member named at the end of this letter if they would like to access their information.

All data collected will be kept in accordance with ECU’s Data Management Policy. Electronic data will be stored on a secure Microsoft SharePoint site provisioned by ECU’s IT Services and physical records will be stored as required in ECU’s Records Management Policy. The data will be retained for a period of seven years and destroyed, if appropriate at the end of the retention period. Data will be de-identified when stored and at the end of the retention period, the data will be destroyed, if appropriate under the State Records Act.

There are no foreseeable risks associated with participation in this research project.

We will advise participants of the outcomes via email communication. We also intend to publish our results in research journals and present them at research conferences locally, nationally, and internationally. Participants' name or any other identifying information will not be included in any of the publications or presentations.

This research project will not start until we sought the approval of Edith Cowan University’s Human Research Ethics Committee under the National Health and Medical Research Council’s National Statement on Ethical Conduct in Human Research 2007 (Updated 2018).

**Data Collection Dates (when undertaking any data collection)**

**01/03/2022 to 02/02/2032**

**Elements 5, 6, and 7**

**Communication of Research Findings or Results to Participants; Dissemination of Project Outputs and Outcomes; After the Project**

Final Report / Report to Funding Body

Peer Reviewed Publication

Lay person summary (e.g., Brochure / Report)

Public presentation

Media release

Summary provided to participants

Conference presentation

Debriefing of participants

Other

**Risk**

**Physical Risks**

Tai Chi/Qigong exercise is safe and there are no foreseeable risks associated with participation in this research project.

**Psychological Risks**

There are no foreseeable risks associated with participation in this research project.

**Social Risks**

No foreseeable social risks associated with participation in this research project.

**Economic Risks**

No foreseeable economic risks associated with participation in this research project.

**Legal Risks**

No foreseeable legal risks associated with participation in this research project.

**Environmental Risks**

No foreseeable risks associated with participation in this research project.
